# Global health ethics: an introduction to prominent theories and relevant topics

**DOI:** 10.3402/gha.v7.23569

**Published:** 2014-02-13

**Authors:** Greg Stapleton, Peter Schröder-Bäck, Ulrich Laaser, Agnes Meershoek, Daniela Popa

**Affiliations:** 1 Department of Health, Ethics and Society, CAPHRI School for Public Health and Primary Care, Faculty of Health, Medicine and Life Sciences, Maastricht University, Maastricht, The Netherlands; 2 Department of International Health, CAPHRI School for Public Health and Primary Care, Faculty of Health, Medicine and Life Sciences, Maastricht University, Maastricht, The Netherlands; 3 Faculty of Health Sciences, Bielefeld University, Bielefeld, Germany

**Keywords:** global health, world health, justice, health inequalities, international ethics, bioethics

## Abstract

Global health ethics is a relatively new term that is used to conceptualize the process of applying moral value to health issues that are typically characterized by a global level effect or require action coordinated at a global level. It is important to acknowledge that this account of global health ethics takes a predominantly geographic approach and may infer that the subject relates primarily to macro-level health phenomena. However, global health ethics could alternatively be thought of as another branch of health ethics. It may then relate to specific topics in themselves, which might also include micro-level health phenomena. In its broadest sense, global health ethics is a normative project that is best characterized by the challenge of developing common values and universal norms for responding to global health threats. Consequently, many subjects fall within its scope. Whilst several accounts of global health ethics have been conceptualized in the literature, a concise demarcation of the paradigm is still needed. Through means of a literature review, this paper presents a two-part introduction to global health ethics. First, the framework of ‘borrowed’ ethics that currently form the core of global health ethics is discussed in relation to two essential ethical considerations: 1) what is the moral significance of health and 2) what is the moral significance of boundaries? Second, a selection of exemplar ethical topics is presented to illustrate the range of topics within global health ethics.

Global health ethics is a relatively new term used to conceptualize the process of applying moral value to health issues that are usually characterized by a global level effect or require action coordinated at a global level. When we discuss ‘global health’ we refer to a phenomenon that is occurring ‘where determinants of health or health outcomes circumvent, undermine or are oblivious to the territorial boundaries of the state and this beyond the capacity of individual countries alone to address through domestic institutions’ (1). Whilst this definition provides a relatively concise account of what phenomena constitute a global health challenge, it does not, however, help us to understand why we should care enough to do something about it. To answer this, one must first explore the scope of global health phenomena to identify potential ethical issues. Once achieved, one can then develop salient moral arguments for or against potential action (including inaction). We consider this process in its entirety to be ‘global health ethics’.

It is important to note that this account of global health ethics takes a predominantly *geographic* approach, which infers that global health ethics relates primarily to large-scale, or macro, health phenomena. In this sense, we could view global health ethics as being concerned primarily with pandemics, natural disasters, poverty, and other phenomena that effect large populations. However, this is not the only approach to global health ethics. It may also refer to health-related topics in themselves through a *content* approach. This type of approach considers both macro-level and micro-level (e.g. clinical research, doctor–patient interactions, patient choice, etc.) health phenomena but conceptualizes these into discrete health-related subjects. Such an approach would view global health ethics as another ‘branch’ of health ethics like that of medical, clinical, or public health ethics (2). Yet, in its broadest sense, global health ethics is a *normative* project. In their description of these approaches, Hunter and Dawson (2) consider normative global health ethics to be a distinct subject in itself. The pioneers and prominent proponents of global health ethics, Benatar, Daar, and Singer (3), argue that before a new global mindset can be addressed a common set of principles to deal with global health threats must be agreed. Hunter and Dawson (2) consider this to be the ‘normative project’ of global health ethics. For them, the normative approach is conceptualized best by Benetar et al.'s (3) challenge of establishing common values that facilitate global health ethics in being ‘committed and engaged in identifying global wrongs related to health and seeking to have them redressed’ (2).

Global health ethics is still a relatively new subject, and health-related issues have not always been framed within its paradigm. This is partly due to its infancy but also due to the plurality of debate that inevitably occurs within such a broadly relevant field. The endeavor to develop a robust ethical framework to apply to issues of global health has been a long and complex process. Recently, ‘international health’ and ‘international justice’ led the discourse through discussions of international ethics and global health justice (4–6). However, theories developed from within these paradigms were originally inspired by discussions taking place within different academic fields such as ‘political philosophy’ (7, 8). Some academic fields have focused more explicitly on normative global health ethics. Global bioethics and global (public) health have deliberately encouraged pluralistic debate of pertinent topics using a global forum in order to establish universally guiding norms (9–11). However, the emergence of the global health ethics paradigm has also been a political pursuit. The exchange of norms and best practices in response to a practical necessity to address both macro- and micro-level health phenomena (12) has also resulted in the globalization of health policy discourse (13) and, therefore, has helped in developing the global health ethics paradigm. Arguably, such a pluralistic and organic development demonstrates a broad interest in ‘both avoiding the enormous risks of doing harm and encouraging individuals to do what is best given a particular sets of circumstances and constraints’ (12). It might then be argued that this is evidence of progress with Hunter and Dawson's (2) *normative* global health ethics project.

This article aims to provide researchers, policy makers, and decision makers with a basic understanding of global health ethics and of some norms principally discussed within the subject area. Through a review of relevant literature, this article delivers a two-part introduction to global health ethics. First, a brief description is provided of prominent theories used within global health ethics (2, 12). Then a selection of high-profile, exemplar ethical conflicts are outlined in order to provide some paradigmatic insight into the range of topics that fall within the scope of global health ethics.

## Theories of global health ethics

At this point, it is important to reiterate that the diversity of topics discussed under the title of global health ethics has not always been framed within its paradigm or even discussed in relation to the concept. However, current scholars that address the question of what is ‘global health ethics’ systematically refer to a selection of influential ethical theories. Among the most prominent of these are the theories of Peter Singer and Thomas Pogge (2, 4, 6, 12). However, before we discuss these we must first consider two key normative issues for theories of global health ethics: what is the moral significance of health and of boundaries?

## The moral significance of health: equity and justice in health

Within global health, we are typically concerned with differences in health and in determinants of health. In order to identify relevant health phenomena and then assess if these are preferable for global health, we must first compare and contrast them against something. In doing this, we are investigating ‘health inequalities’. However, not all health inequalities are unjust. We see huge diversity in the world, yet, from an ethical point of view it is not clear what we have a moral duty to remedy or how we should go about remedying it. Nonetheless, it can be agreed that health has special moral importance; therefore, health inequalities are also morally significant. ‘Good’ health limits suffering, yet, also enhances our capacity to function and, therefore, extends the range of opportunities open to us in life (14–16). Health justice is principally concerned with reducing unfair and avoidable health inequalities rather than eliminating differences in health states altogether. Therefore, within health justice we are less concerned with inequalities and primarily concerned with pursuing ‘equity’ in health. In other words ‘that everyone should have fair opportunity to attain their full health potential and, more pragmatically, that no one should be disadvantaged from achieving this potential, if it can be avoided’ (17).

## The moral significance of boundaries: cosmopolitan and anti-cosmopolitan views

Another important issue for global health ethics is whether or not geopolitical boundaries have *moral* significance. For cosmopolitans, every person is a ‘world citizen’ (18); therefore, everyone has a moral duty to assist those in need regardless of their proximity or their nationality. To cosmopolitans, the global geopolitical environment has no moral relevance other than for its potential influence on the certainty of achieving a preferred outcome. In contrast, anti-cosmopolitans argue that morality is ‘local’ and specific to cultures (19). They reject an impartial view of morality under the assumption that universal norms are unlikely to be agreed upon in light of the plurality of global cultures. However, they can be agreed upon within communities; consequently, communities and their ‘members’ (e.g. citizens or compatriots of national states) have a special, morally significant, relationship with one another. Yet, these positions can be critically discussed further. For example, Hunter and Dawson (2) describe cosmopolitanism in terms of ‘moral’ or ‘political’ views. They consider moral cosmopolitanism to stress that ‘moral judgments and obligations are universal and impartial in nature’ (2), whilst political cosmopolitanism, they argue, acknowledges a role for geopolitics but focuses on strengthening just (global) institutions and weakening state politics (2). Further positions within anti-cosmopolitanism can also be seen such as a ‘realist’ or ‘pluralist’ notion of anti-cosmopolitanism. Realists argue that national boundaries limit ethical considerations. For them, global society is an anarchical system in which one must prioritize national interests first. Therefore, moral considerations are limited by the state's culture. However, pluralists take a much weaker stance. For them, diversity and pluralism, at a global level, are not necessarily harmful to national interests. Therefore, whilst pluralists also consider morality to be local, they may also recognize some moral worth in coexisting (19).

But what does this mean for global health ethics? At first glance one might assume that the anti-cosmopolitan stance is much less demanding towards global health action. We might even believe that this position is selfish. However, anti-cosmopolitan views of morality do not necessarily negate the possibility of global level health action. For example, aid may still be provided to other countries that are suffering under the premise that this protects national interests in the long run. Furthermore, nation states may also place more value in acting upon the imperfect duty of beneficence (e.g. charity). Therefore, aid may still be given to global health action, at least when this does not conflict with national interests.

## A substantive approach to justice: Singer and preference utilitarianism

The key concerns in preference utilitarianism are that there is a moral imperative to maximize happiness and to avoid suffering. Distance or proximity are just ‘geographical contingencies’ (20) and as such are morally irrelevant. Within Singer's theory of preference utilitarianism, the more affluent should support the least privileged whenever this can be achieved without costing anything of comparable moral significance. Therefore, if we have the capacity to help others at minimal (relative) cost to ourselves, then it is our moral duty to help, regardless of their proximity to us. Singer (4) builds his theory from a premise he assumes everyone agrees upon, namely, that suffering and (premature) death are bad or are at least not preferred. As is common to utilitarian theory, Singer's approach prioritizes the avoidance of suffering and also considers that greater aggregate suffering is of higher moral significance. Thus, his basic principle for global health ethics (although not termed ‘global health ethics’) states that: if we can prevent something bad without having to sacrifice something of equal moral importance, then we are *morally obliged* to act.

This cosmopolitan principle takes no account of proximity. For preference utilitarianism, boundaries of communities or nation states are morally irrelevant. As Singer has consistently argued: ‘the development of the world into a ‘global village’ has made an important … difference to our moral situation’ (4), something that is perhaps more apparent within the last decade. Whilst Singer acknowledges that there are psychological differences (e.g. feeling less guilty about suffering that is less obvious), he dismisses these as immaterial from the moral perspective. In summary, one is obligated to redistribute a reasonable amount of one's affluence to lessen the suffering of others as long as this does not *endanger* oneself or one's family.

## A procedural approach to justice: Pogge and liberal cosmopolitanism

Unlike Singer's substantive approach, Pogge's (6) theory takes greater account of geopolitics using a procedural approach to justice. Within his theory, rights are derived from within the structure of global society and from its institutions, which he suggests are unjust. Pogge argues from a rights perspective, which distinguishes between negative and positive rights and duties. Negative rights (or duties) are concrete and always true whereas positive rights (or duties) to help are weaker but more encompassing. Pogge's negative moral duties extend only so far as achieving a just society; consequently, inequalities may still remain. However, residual inequalities may still demand action but only under (weaker) positive duties (e.g. charity).

Pogge takes an egalitarian view of morality: ‘the global poor have done nothing to deserve their position – in fact, most of them are children’ (6). His perspective treats individuals in developed or developing countries as moral equals. However, the unequal suffering experienced by developing nations as a result of unfair historical exploitations by the most privileged (e.g. resources attained unfairly) is unjust. Whilst it can be argued that it is unfortunate but not unjust for there to be ‘losers’ within global society, Pogge claims that the causal relationship between world order and continuing harm to developing nations undermines this argument (4, 21). He suggests that manipulation of the global structure has advantaged, and continues to advantage, the affluent whilst disadvantaging the underprivileged and, therefore, perpetuates global inequity. However, unlike Singer's preference utilitarianism, where there is a moral duty against all individuals to act against any (relative) inequality (as long as there is limited risk to oneself), Pogge assigns only limited duties that focus on reforming unjust global institutions: ‘by continuing to support the current global order and the national policies that shape and sustain it without taking such compensating action, we share a negative responsibility for the undue harms they foreseeably produce’ (4). In summary, whilst we have a very clear negative duty to avoid harm and not to take advantage (unjustly) of the least privileged, this is perhaps less stringently applied in terms of individual agency, which requires only negative duties of pursuing reform and positive duties of aid.

## Ethical conflicts: a review of ethical topics

The most typical public health ethical conflict is in deciding upon how to balance the needs of ‘the many’ against the rights of ‘the individual’. Classic examples of this dilemma are who should be saved if not everyone can be saved and how can an individual's privacy and liberty be respected whilst still protecting and promoting the health of others (11)? When addressing these questions, ‘trade-offs’ inevitably occur; however, in order to ensure that these are fair and just, one must be able to assess the duties and rights of all the parties involved. Only then can one ascertain a balance that delivers ‘just’, yet, practical solutions. Singer's and Pogge's theories (amongst others) can help us to frame these questions. In practice, global health issues are complicated, are numerous, and have many parameters that are ethically relevant. Identifying these parameters is as much a task of ethics as is delivering just solutions.

Some of the most high-profile ethical issues in global health are the ‘brain drain’, inequitable distribution of resources (e.g. primary goods such as water, food or housing), gender or race inequality (discrimination), health system financing, the spread of infectious diseases, poverty, etc. (3). However, global health issues are sensitive to a range of phenomena (e.g. economic crisis, demographic changes or migration etc.) that can significantly complicate their resolution (3). For example, how does one balance the free movement of health care professionals against maintaining the effective provision of regional health care (22)? Scientific progress has also complicated issues by enhancing our understanding of the etiology of health states. Such progress may simultaneously enhance the capacity of health care systems (and other institutions) with new approaches to prevent, treat, or promote health. These developments indirectly affect our understanding of who is responsible, or accountable, for people's health.

How these influences are identified and accounted for in global health ethics is the responsibility of researchers, policy makers, and decision makers who must address relevant issues within their role. The following represent a selection of classical, high-profile global health concerns that have been described in order to highlight the diversity of global health ethics and provide some paradigmatic insight into the subject.

## Infectious disease research, the 10/90 gap and the Health Impact Fund

Whilst infectious diseases represent a considerable global burden of disease (23), the ethical challenge is that much of this burden would be preventable if greater support was provided to regions with greatest need. Considerable knowledge about prevention and treatment exist; however, this is simply not made accessible to much of the developing world (21). What is most striking is not that it would be possible to help by making existing treatments more accessible but that even medical research in the global community can be inequitable (24). Prior to the 1990s, it was estimated that less than 10% of worldwide investment in health research was dedicated to health problems associated with 90% of the global burden of preventable mortality. Potentially preventable diseases that are severe if untreated, have previously received comparably little attention in the research community because it has been the ‘better off’ funding research for their own benefit (i.e. research for those who can pay most and not for those in greatest need) (24).

Even if research could be focused on global health impact, new medical technologies would still need to be made more accessible to those that require them, and it is often the poorest that are in greatest need. Hollis and Pogge (25) argue that reforming the global system of finance and coordination of medical research would incentivize more equitable research and facilitate more equitable access to treatments. They argue that the global patent regime as determined by the Agreement on Trade-Related Aspects of Intellectual Property Rights (TRIPS) elevates the price of medicines and biases research. The TRIPS scheme, which protects patents and incentivizes research, unfairly favors medical research that targets the health concerns of developed nations. In order that health research becomes more reflective of actual need, Hollis and Pogge (25) recommend implementing a new system: the Health Impact Fund (HIF). The proposed HIF facilitates a more open research market that will prioritize research of medicines with greatest global health impact. Yet, it is financed in a way that also satisfies the moral duties of preference utilitarianism. Participation funds are provided to the HIF from 0.03% gross national income. Therefore, nations will contribute (approximately) according to their level of affluence. Funding is then granted by the HIF to pharmaceutical companies based only on the ‘global health impact’ of their medicines. However, to gain access to the HIF funding, pharmaceutical companies must agree to sell their products at manufacturing prices. Thus, access to more helpful medicines will become more equitable and, therefore, act to reduce global inequalities in health.

## Research in low-income countries and informed consent

The need to refocus medical research towards addressing the severe inequalities in the global burden of disease is evident. Medical research in low-income countries can be hugely beneficial to participants (e.g. complementary health care, compensation, access to state of the art medicines, etc.), yet, how can we ensure that research is conducted ethically and in a way that protects participants, especially when they might be the least privileged and most vulnerable members of global society? Several ethical standards have been proposed over the last century, particularly when reflecting upon the historic atrocities of immoral medical research in Germany (26). However, there has been serious ethical misconduct using low-income populations as recently as the mid-1970s. The experiences of the Tuskegee syphilis study present a disturbing example of observational research that exploited, and harmed, vulnerable populations. The research, involving an Afro-American population with syphilis, was continued without offering an effective medicine to participants once it was available. Participants were kept ignorant of the study's goals and of alternative treatment options provided outside the study. Even though this study took place several decades ago within the USA, the example illustrates how global health research can exploit, disrespect, and put at risk, the most vulnerable and socially disadvantaged populations in society (26).

This example is also illustrative of the scope of global health ethics. The primary ethical conflict pertains to the conduct of medical research involving humans and is predominantly focused at the level of individual agency. As such, the issue may be initially thought of as predominantly bioethics, but will also be relevant to global health ethics through a content approach. However, the issue of how medical research involving humans is conducted is also pertinent to global health ethics through a geographic account of the subject. This is perhaps most obviously the case when stakeholders and research participants are located in different countries and demonstrate a substantial difference in levels of vulnerability and social privilege. The report delivered by the Nuffield Council on Bioethics (27) outlined potential ethical conflicts of research conducted by developed countries within developing countries. The report details an ethical framework that emphasizes moral duties to alleviate suffering, respect participants, to remain sensitive to cultural differences and not to exploit vulnerable populations. These duties are also reflected in the recommendations for the conduct of biomedical research involving humans by the Council for International Organizations of Medical Sciences (CIOMS) in collaboration with the WHO (28), which delivers a set of ethical guidelines based upon the three core principles: 1) respect for persons a) respect for autonomy and b) protection of persons with impaired or diminished autonomy; 2) beneficence and 3) justice. Such recommendations have also been mirrored within the more recent Tri-Council Policy statement of 2010 (29), whereby the core ethical principles for health research involving humans are respect for persons, concern for welfare and justice. These principles form benchmark criteria for independent research ethics committees and, therefore, influence medical research internationally. Such criteria may then, ultimately, be understood in terms of normative global health ethics.

Within the Nuffield Council's report, special emphasis is placed on attaining informed consent from potential study participants and, where appropriate, from their respective communities or family members. Yet, how can one know that subjects fully understand the study and its risks and are, therefore, fully ‘informed’? Poor levels of education and health literacy present the most obvious difficulties for attaining informed consent; furthermore, these are often issues that more frequently burden vulnerable and underprivileged populations (30). However, ethical issues of informed consent may be much more subtle (31). Zaman and Nahar (32), for example, noted that even the presumed universality of research ethics is ‘Euro-American’ and often unrecognized within other cultures. Consequently, and quite paradoxically, even when trying not to exploit participants, one might still suffer from an ‘ethnocentrism’ before research has even begun. In addition, some of the cultures that do not understand the ‘Euro-American’ concept of ‘research’ may not even speak a language in which it can be easily communicated. As Zaman and Nahar (32) wrote: ‘We found it difficult to make the villagers understand what ‘research’ is, as there is no equivalent term for it in rural Bangladesh. The formal Bengali word derived from the Sanskrit language has a meaning connected with ‘finding a lost cow’’ (32).

## Teaching global health ethics

Education of global health students is a ‘meta-topic’ within global health ethics that is highly relevant for achieving sustainable capacity building of global health ethics competence. Pinto and Upshur (12) describe that nowadays more and more students, including medical students, are attracted to global health. Frequently they are motivated by a sense of beneficence; however, sometimes their motive is self-interest (e.g. careers opportunities in global health). Pinto and Upshur (12) argue that an ethical education is paramount to any global health education: ‘without appropriate training students are unprepared to face ethical dilemmas in global health and risk causing harm to patients, research subjects, and communities. Teachers and institutions have a responsibility to provide training in ethics as an essential precursor to global health work’. They argue that students must be equipped with the correct ‘tools’. In other words, an ethical framework that differs from the classic bio- or medical ethics. This framework should focus on typical global health work in developing countries and encourage humility amongst medical students, and the increasing number of non-medical students, studying global health: students should not become ‘medical tourists’ but be aware of their limitations, their involvement in the developing countries, and the harm that can result with even the best of intentions.

## Discussion

This paper has presented a mix of exemplary morally relevant issues in the context of two prominent theories of global health ethics. The example ethical conflicts discussed highlight the breadth of global health ethics and the difficulty in demarcating a global health ethics paradigm from other forms of health ethics. Whilst Hunter and Dawson (2) are able to conceptualize global health ethics by three different approaches (geographic, content, and normative), it is a much harder project to outline the broad range of ‘borrowed’ ethics that currently form the core of the global health ethics paradigm. Global health ethics bridges theories of international ethics with traditional branches of health ethics and, therefore, can be applied to both macro- and micro-level health phenomena. Both Singer and Pogge recognize that global inequality in suffering has moral significance. If we consider poor health to be indicative of suffering, then special moral importance can also be applied to inequalities in global health (14). However, how Singer and Pogge assign morality to actions differs substantially. Both theories stress a global perspective that is perhaps most useful for discussing health issues through a geographic approach to global health. Consequently, these theories are less applicable to the full range of health-related issues that are characterized by individual agency. For this, global health ethics may defer to norms derived from bioethics or medical ethics, etc. The principles outlined in the Nuffield Council on Bioethics 2002 report (27) are considered to be ‘bioethics’ or ‘research ethics’, etc. However, their development into universal norms for international health research falls directly in line with Hunter and Dawson's (2) normative approach to global health ethics.

## Conclusion

It must be understood that this paper has delivered a cursory introduction to global health ethics and is only intended to help researchers, policy makers, and decision makers understand the different accounts of global health ethics and become aware of the most prominent theories that currently form the paradigm's core ethical framework. ‘We cannot escape ethical dilemmas. When confronted, our responsibility is to reason our way through them, identify the best options (or the least bad ones), and to act according to our best judgment’ (11). In their description of the role of ethics, Wikler and Cash are deliberately non-specific as ethics can be applied to any phenomenon. Global health is no exception. Decisions made in response to large-scale, or macro, health phenomena, such as pandemics, have the potential to avoid significant harm. However, such harm is often the cumulative effect of phenomena occurring at the level of individual agency; therefore, micro health phenomena are also pertinent to global health ethics. Whilst it is the *duty* of ethicists to develop ideals for global health, it is, however, our *hope* that applying strong ethics to decision making at all levels achieves a more complete, and universally accepted, ethical framework for global health ethics.
